# Author Correction: Trait mediation explains decadal distributional shifts for a wide range of insect taxa

**DOI:** 10.1038/s41467-025-64720-4

**Published:** 2025-10-01

**Authors:** Yoann Bourhis, Alice E. Milne, Chris R. Shortall, Björn Beckman, Dan Blumgart, Rowan Edwards, Luke C. Evans, Chris W. Foster, Richard Fox, Marc S. Botham, Clare Rowland, Stuart Roberts, Martin C. D. Speight, Chris Hassall, William E. Kunin, James R. Bell

**Affiliations:** 1 Net Zero Resilience Farming, Rothamsted Research, West Common, Harpenden, UK; 2 British Trust for Ornithology, The Nunnery, Thetford, UK; 3 Hymettus Ltd, Midhurst, UK; 4 School of Biological Sciences, University of Reading, Whiteknights campus, Reading, UK; 5 Butterfly Conservation, East Lulworth, Wareham, UK; 6 UK Centre for Ecology and Hydrology, Crowmarsh Gifford, Wallingford, UK; 7 UK Centre for Ecology & Hydrology, Library Avenue, Bailrigg, Lancaster, UK; 8 School of Agriculture, Policy and Development, University of Reading, Reading, UK; 9 School of Natural Science, Trinity College, Dublin, Ireland; 10 School of Biology, University of Leeds, Leeds, UK; 11 School of Life Sciences, Keele University, Keele, Newcastle, UK

**Keywords:** Conservation biology, Phenology, Machine learning, Biodiversity

Correction to: *Nature Communications* 10.1038/s41467-025-63093-y, published online 30 August 2025

In this article there was an error with Figure 5 in which some of the text characters did not get rendered correctly. The original article has been corrected.

