# Supplementary material for: Trait mediation explains decadal distributional shifts for a wide range of insect taxa
Source: Nat Commun. 2025 Aug 30;16:8131. doi: 10.1038/s41467-025-63093-y (PMC12398571; doi:10.1038/s41467-025-63093-y)
Supplement: Supplementary file 1 — Supplementary Information [file 41467_2025_63093_MOESM1_ESM.pdf]

Supplementary Information

Trait mediation explains decadal distributional shifts for a wide range of insect taxa

Bourhis *et al.*

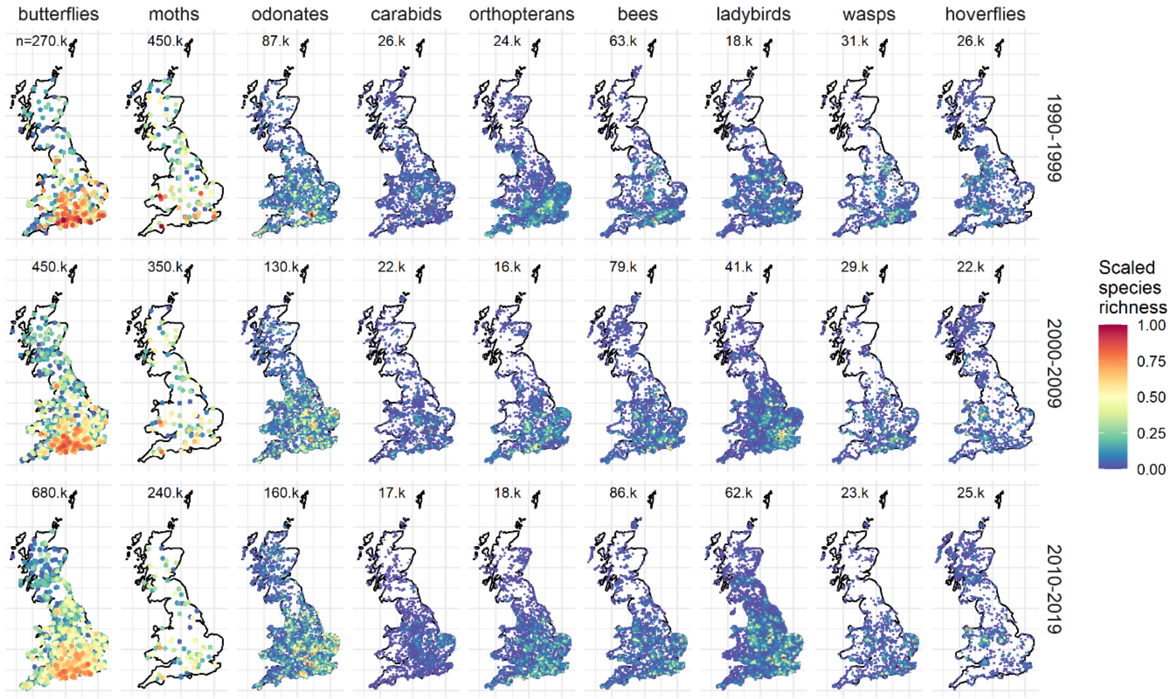

Fig. S1: Distribution of sample points for the nine insect groups considered here. Butterflies and moths are presence-absence data set, while the rest are presence-only aggregated to a contingency table over a 1 km x 1 km grid. The number of observations per insect group and decade is reported on the top left-hand corner of each panel. For the butterflies and moths, both presences and absences are counted as observations, while only presences are counted as such for the other groups. The outline of Great Britain comes from the R package ggplot2.

Supplementary Note 1

Fig. S2 illustrates the model predictive performance for each insect group. It addresses matters related to (i) data leakage, (ii) the tuning of one hyperparameter (the exponent used in species-specific class weights) and (iii) the efficiency of SHAP at highlighting a minimal yet sufficient set of key drivers.

The wide bars (*20 bags*) show the performance of the 20-bag ensembles discussed throughout the paper. The middle four bars (*3 bags*) show the performance for a 3-bag ensemble, they serve as a benchmark for the following four bars. The following four bars (*3-block CV*) show the

performance for cross-validation with a split made of spatiotemporally contiguous blocks.

The difference in performance between the middle four bars and the right-hand four bars quantifies the part of learning attributable here to data leakage. This is caused for example by two extremely similar samples (i.e. very close in the input and output spaces) falling on each side of the train/test split, resulting in an overestimation of testing performance. Data leakage varies from group to group but remains limited overall.

Next, the colours mark the weight exponent, with orange for the linear species-specific class weights and blue for the square root species-specific class weights. The square root weights are softer in correcting for the imbalance between presence and absence. We have found that square root class weights work better for presence-absence (PA) data, while linear weights are better for presence-only (PO) data. This is likely due the zeros being much more informative in PA than in PO, hence softer weights allow them to have greater influence in the learning. The weight exponent was selected based on the *3-block CV* performances. Note that, although the odonates and orthopterans are classified as PO data sets here (for simplicity), their reduced number of species as well as the fact that many of their NBN records are in fact *full list* records (i.e. PA data), there is no clear winner in terms of exponent.

Finally, the black outline and transparency of the bars mark the number of drivers used. The transparent bars show the performance of models trained with half the number of drivers, keeping only the ones highlighted in Fig. 3. The drop in performance when doing so quantifies the part of learning attributable to the 15 other drivers. We show here that the drop is very limited, hence supporting the driver selection made by the SHAP method.

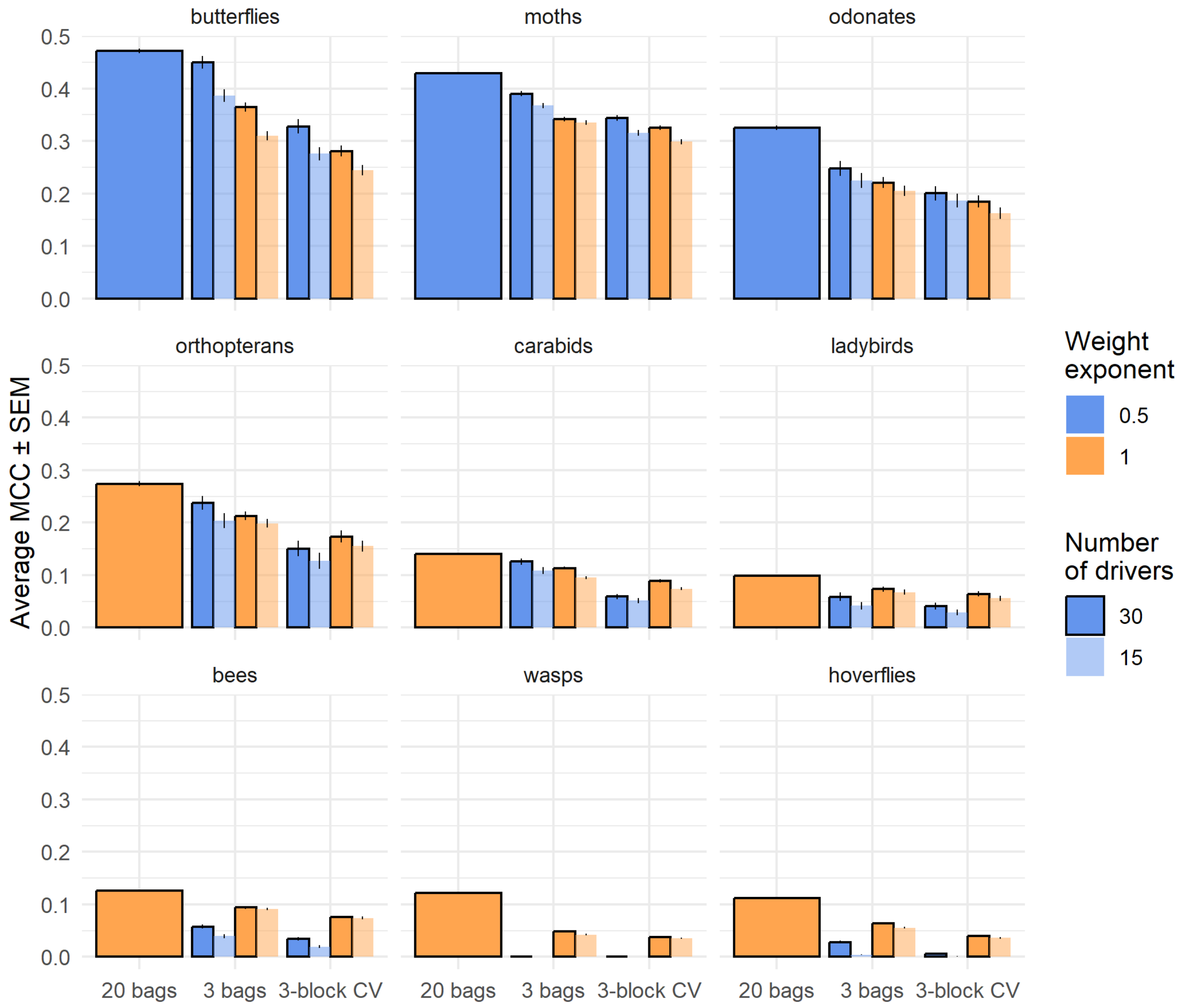

Fig. S2: Comparing the prediction performances with (1) using linear and square root weights as species-specific class weights, as well as (2) when the set of drivers is halved by selecting only the most important ones highlighted in Fig. 3. Performance (Average Matthews Correlation Coefficient, MCC ± Standard Error of the Mean) is shown for the full ensemble of 20 bags, a smaller ensemble of 3 bags, and a block cross-validation made of 3 folds within 12 blocks of spatiotemporally contiguous samples.

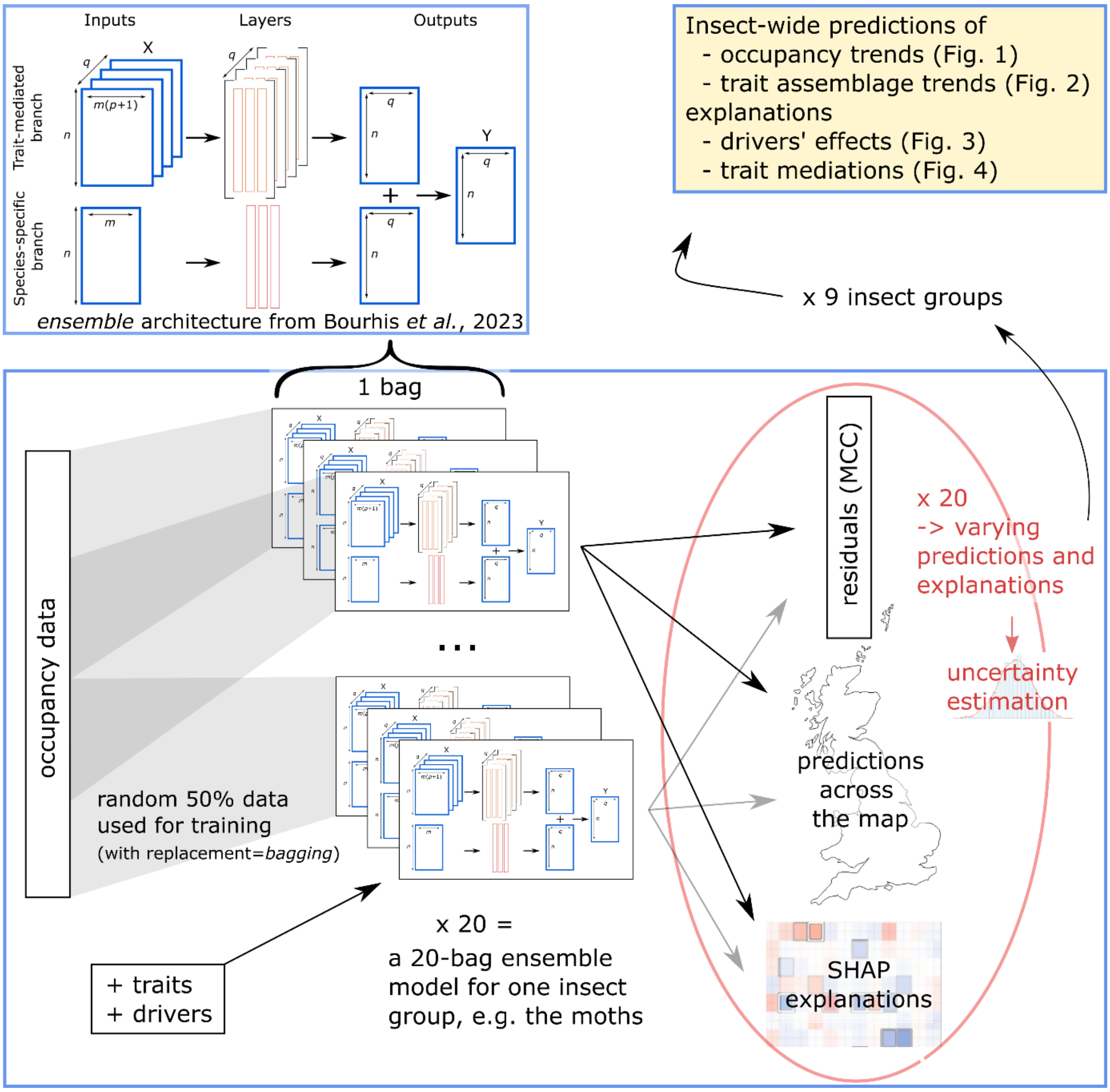

Figure S3: Schematic view of the ensembling process. For a given insect group, the process consists in training 20 different models with slightly varying input data. The input variation comes from sampling (with replacement) the occupancy data. This process is called bagging, short for bootstrap ensembling. Each one of those 20 models or bags then produced slightly varying outputs (predictions and SHAP explanations), from which uncertainty estimates can be produced at the ensemble level. By doing this for the 9 insect groups of the study, we produce the insect-wide predictions and explanations that are presented in this study. In the top left-hand corner box, the input (X) and output (Y) dimensions are: n*, the number of samples;* m*, the number of drivers;* p*, the number of traits; and* q*, the number of species. The outline of Great Britain comes from the R package ggplot2.*

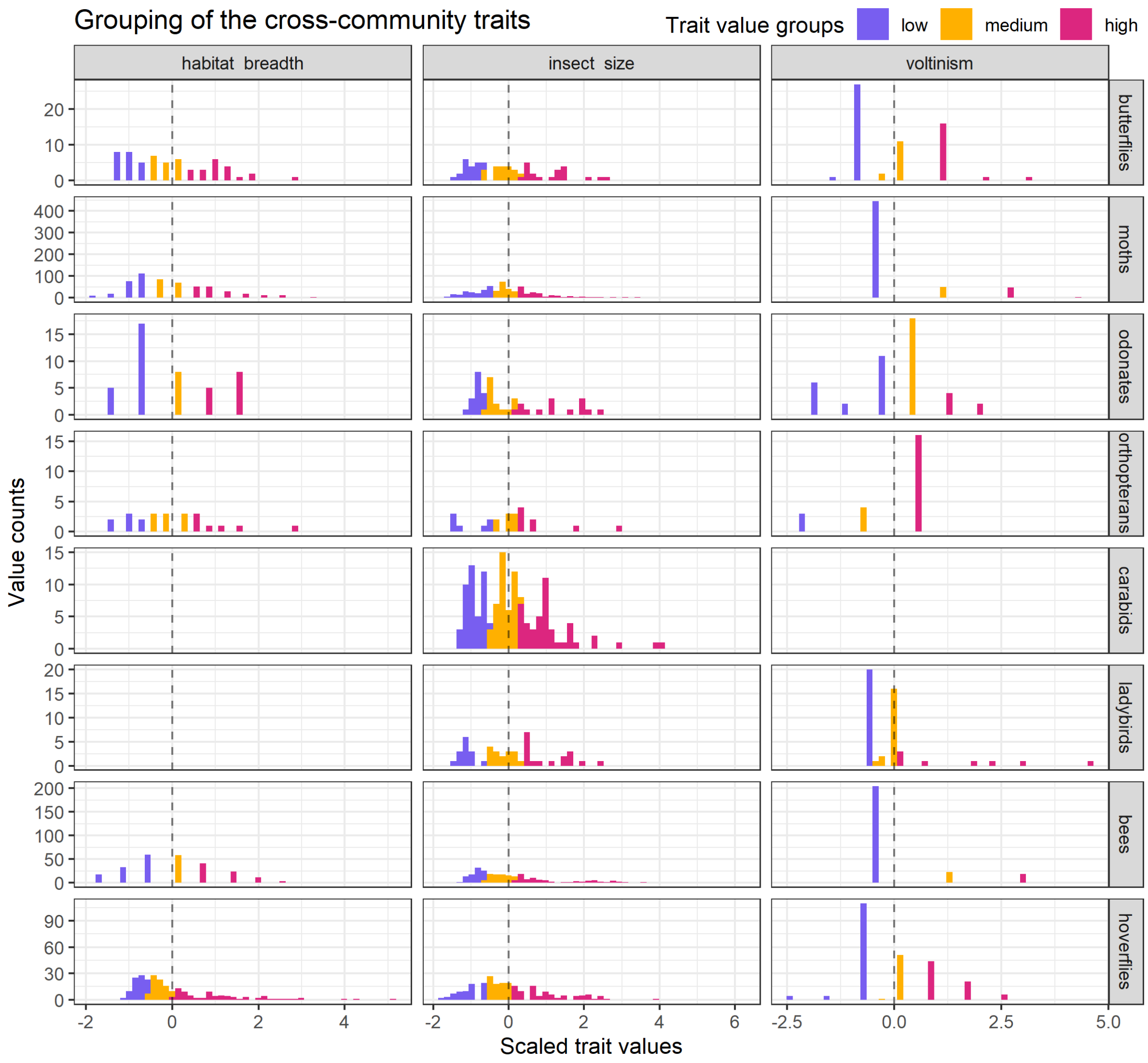

Fig. S4: Distribution of trait values across taxa and illustration of their cutting into 3 groups (low, mid and high). The trait values are scaled (reduced centered). The cuts are made to result in 3 groups of as much as possible equal sizes, while never separating into different groups species of identical trait values (which happens normally with quantiles). Note that carabids have only one of the cross-taxa traits informed, while ladybirds have only two.

Table S1: The different environmental drivers selected for our models. All are informed yearly at 1 km^2 scale. The land cover related drivers are derived from 25 m x 25 m pixels attributed with 21 land cover classes or 10 agregated classes (UKCEH land cover maps). The compositional metrics are derived on the 10 agregated classes, while `lc_diversity` *and* `lc_contiguity` *are derived on the 21 classes. The weather related metrics is composed of the BIOCLIM19 set of metrics, derived from the Had-UK grid with monthly and 1 km^2 resolution. This set of drivers results from dropping correlated drivers having more than 85% correlation across the whole of Great Britain.*

landscape	`%arable`	Proportion of arable 25m pixels within the 1km pixel
	`%broadleaf`	Same with broadleaf woodland
	`%coast`	Same with coastal areas
	`%conifer`	Same with coniferous woodland
	`%imprv_grs`	Same with improved grassland
	`%mountain`	Same with mountain, heath and bog
	`%seminat_grs`	Same with seminatural grasslands
	`%urban`	Same with urban cover
	`lc_contiguity`	Average proportion of neighbouring 25m pixels that share the same land cover
	`lc_diversity`	Shannon diversity of land cover
topology	`elevation`	Terrain elevation
	`slope`	Slope of the terrain
	`aspect`	Orientation of the slope
	`dist_to_sea`	Distance to the closest coast
	`river_dens`	Density of the freshwater network
climate	`temp_avg`	Average temperature throughout the year
	`temp_diu_ran`	Temperature diurnal range
	`temp_dry_qtr`	Average temperature throughout the driest quarter of the year
	`temp_max`	Maximal temperature
	`temp_min`	Minimal temperature
	`temp_season`	Temperature seasonality
	`temp_wet_qtr`	Temperature of the wettest quarter of the year
	`isotherm`	Isothermality
	`rain_sum`	Cumulated rainfall throughout the year
	`rain_min`	Cumulated rainfall of the driest month
	`rain_season`	Rainfall seasonality
	`rain_warm_qtr`	Rainfall during the warmest quarter of the year
	`past_rain_sum`	Cumulated rainfall throughout the previous year
	`past_temp_avg`	Average temperature throughout the previous year
	`wind_avg`	Average wind speed throughout the year

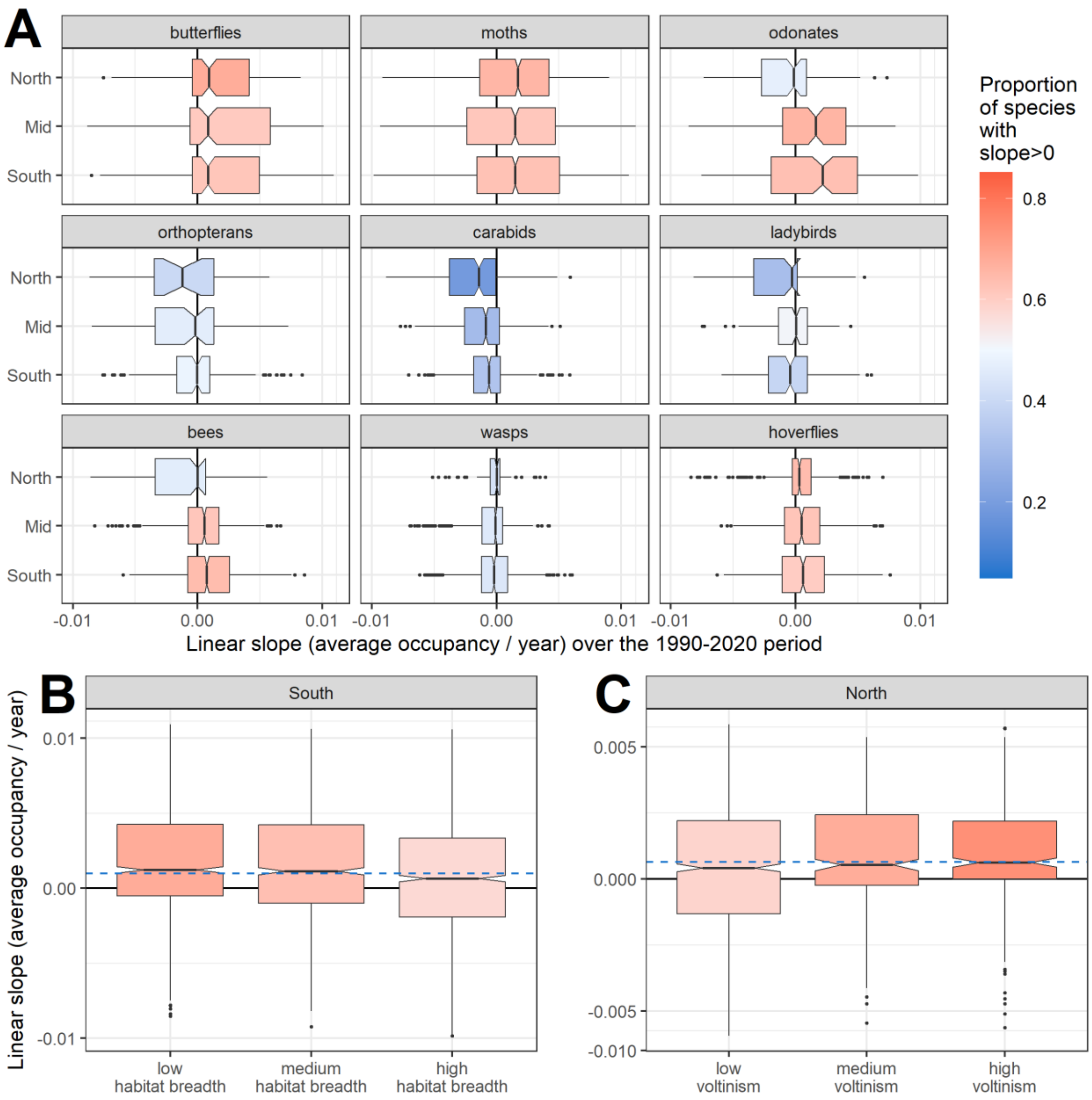

*Figure S5: Distributions of the species linear slopes in average occupancy across 1990-2020. (**A**) Slopes are shown per regions and taxonomic groups. (**B**) Slopes in the south shown per group of habitat breadth trait value, with low habitat breadth species showing higher slopes than high habitat breadth species, hence explaining the decrease in habitat breadth in the south reported in Fig. 2. (**C**) Slopes in the north shown per group of voltinism trait value, with high voltinism species showing higher slopes than low voltinism species, hence explaining the increase in voltinism in the north reported in Fig. 2. The boxes show the median of the distribution as well as the first and third quartiles, extended with lines of +/- 1.5 inter-quartile range (IQR). Data points (black dots here) located outside this range are outliers. The notches extend 1.58 * IQR / sqrt(n) around the median. The blue dashed line is a visual aid for comparing the boxplot notches whose mutual exclusion suggests significantly different medians. Here, the medians appear significantly different (for the low and high groups) in **B** but not in **C**.*

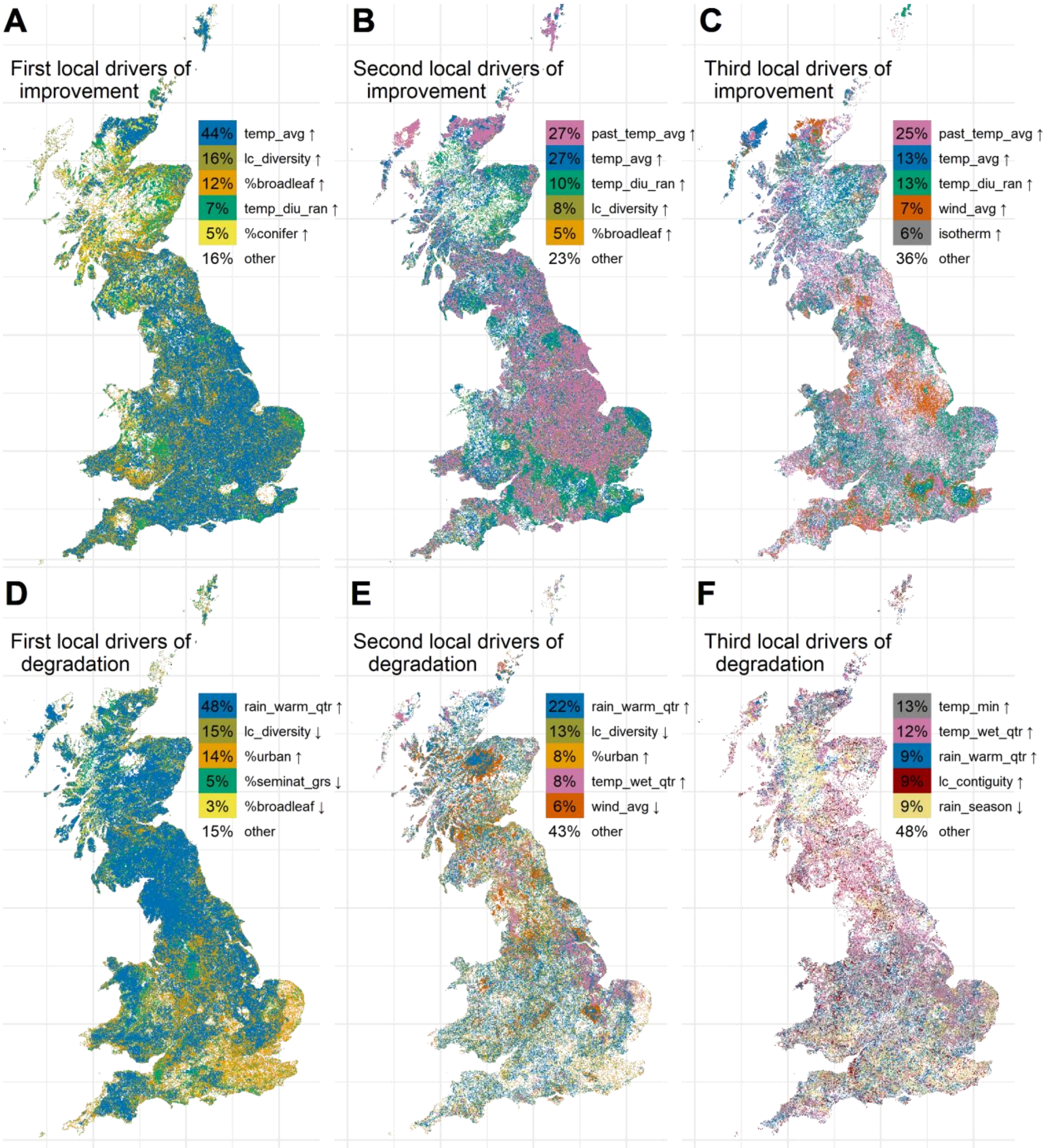

Fig. S6: Maps of the main local causes of insect occupancy increases (top row) and decreases (bottom row). The percentage values in the legend indicate the proportion of the map covered by each of the colours. For example, ***D*** *shows that increases (↑) in the proportion of urban cover and decreases (↓) in the proportion of broadleaf forest are the first causes of degradation in 14% and 3% of the map respectively. For 8% of the map, the secondary cause of degradation is increases (↑) in the proportion of urban cover (**E**).*

Supplementary Note 2

Here is a simulation experiment that illustrates the effects of bagging and species-specific class weights when estimating temporal trends of virtual species. Here, the virtual species are given a diversity of spatiotemporal distributions (through spatiotemporal gaussian random fields) that are subsequently sampled. The sampling occurs either as full-list records or as individual observations, resulting respectively in presence-absence and presence-only data. Additionally, the sampling is done with controlled levels of spatial, temporal and taxonomic biases, including no bias at all.

For a given overall sampling effort needed to generate a data set, the absence of bias means that every year, location and species are given an equal sampling effort. Temporal bias involves the latest years of the 30-year simulation having double (or half) the sampling effort given to the early years, with a linear increase (or decrease) in between. The spatial bias is the same but across latitude. The taxonomic bias involves some species being looked for up to twice (or ten times) as much as others.

The generated data sets are subsequently used to train binomial generalised additive models (GAMs[1]). During training, some GAMs use species-specific (linear) class weights as sample weights, some don't. Those GAMs then produce predictions across the space-time grid at a large number of random (unbiased) times and locations. Those predictions are used to derive species-specific occupancy trends.

This experiment shows (Fig. S7) that:

- Models building on presence-only data need species-specific class weights to produce useful predictions of species occupancy trends.
- Increasing sample size reduces uncertainty.
- Using species-specific class weights increases uncertainty significantly because it reduces the effective sample size.

More interestingly, the experiment shows that bagging and species-specific class weights, when used jointly, are an effective mean of training models that produce species-specific occupancy trends with accurate uncertainty estimates.

This supports our study by showing that unstructured data can be made useful and that trading quality for quantity is a valid option, especially now

that opportunistic single-species observations become more widely available.

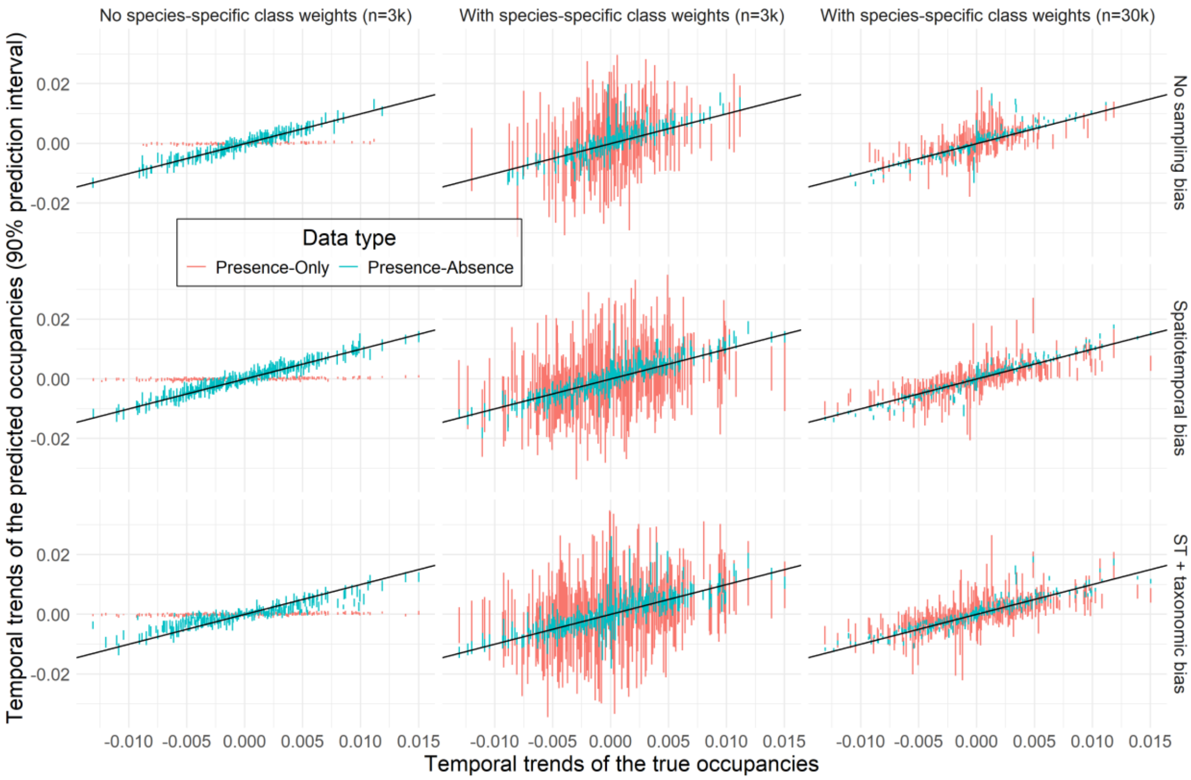

Fig. S7: Agreement between predicted (90% prediction interval error bars) and true occupancy trends (single values) for a set of simulated species. The predictions result from GAMs trained on presence-absence (in blue) or presence-only data (in red), generated without bias (first row), with spatiotemporal (ST) bias (middle row), or both ST and taxonomic biases (bottom row). The black line has a slope of 1, marking identity. The error bars encompassing the black line include the true value of the species temporal trend, marking successful uncertainty estimations. From panels left to right, the uncertainty estimation improves drastically, first through the use of species-specific class weights (columns left to middle) then by increasing sample size (columns middle to right).

Reference

1. Hastie, T. & Tibshirani, R. Generalized Additive Models. *Stat. Sci.* **1**, 297–318 (1986).
