# Supplementary material for: Trait mediation explains decadal distributional shifts for a wide range of insect taxa
Source: Nat Commun. 2025 Aug 30;16:8131. doi: 10.1038/s41467-025-63093-y (PMC12398571; doi:10.1038/s41467-025-63093-y)
Supplement: Supplementary file 2 — Reporting Summary [file 41467_2025_63093_MOESM2_ESM.pdf]

nature portfolio

Corresponding author(s): Yoann Bourhis

Last updated by author(s): Jun 2, 2025

Reporting Summary

Nature Portfolio wishes to improve the reproducibility of the work that we publish. This form provides structure for consistency and transparency in reporting. For further information on Nature Portfolio policies, see our Editorial Policies and the Editorial Policy Checklist.

Statistics

For all statistical analyses, confirm that the following items are present in the figure legend, table legend, main text, or Methods section.

n/a	Confirmed	
☐	☒	The exact sample size (n) for each experimental group/condition, given as a discrete number and unit of measurement
☒	☐	A statement on whether measurements were taken from distinct samples or whether the same sample was measured repeatedly
☐	☒	The statistical test(s) used AND whether they are one- or two-sided<br>*Only common tests should be described solely by name; describe more complex techniques in the Methods section.*
☐	☒	A description of all covariates tested
☐	☒	A description of any assumptions or corrections, such as tests of normality and adjustment for multiple comparisons
☐	☒	A full description of the statistical parameters including central tendency (e.g. means) or other basic estimates (e.g. regression coefficient) AND variation (e.g. standard deviation) or associated estimates of uncertainty (e.g. confidence intervals)
☐	☒	For null hypothesis testing, the test statistic (e.g. F, t, r) with confidence intervals, effect sizes, degrees of freedom and P value noted<br>*Give P values as exact values whenever suitable.*
☒	☐	For Bayesian analysis, information on the choice of priors and Markov chain Monte Carlo settings
☒	☐	For hierarchical and complex designs, identification of the appropriate level for tests and full reporting of outcomes
☒	☐	Estimates of effect sizes (e.g. Cohen's d, Pearson's r), indicating how they were calculated

Our web collection on statistics for biologists contains articles on many of the points above.

Software and code

Policy information about availability of computer code

Data collection	No software was used.
Data analysis	R 4.4.2 and Python 3.10.<br>Online repository for the code: https://gitlab.com/Yo-B/minimal_sdm<br>As well as ZENODO reposiory with DOI: https://zenodo.org/records/15572921

For manuscripts utilizing custom algorithms or software that are central to the research but not yet described in published literature, software must be made available to editors and reviewers. We strongly encourage code deposition in a community repository (e.g. GitHub). See the Nature Portfolio guidelines for submitting code & software for further information.

Data

Policy information about availability of data

All manuscripts must include a data availability statement. This statement should provide the following information, where applicable:

- Accession codes, unique identifiers, or web links for publicly available datasets
- A description of any restrictions on data availability
- For clinical datasets or third party data, please ensure that the statement adheres to our policy

Occupancy data can be queried by contacting the respective monitoring schemes and societies for curated data sets (i.e. UKBMS for butterflies, RIS for moths, BDS for odonates, BWARS for bees and wasps), or downloaded from the NBN atlas for more diverse data collections. For drivers, land-cover data can be queried from

UKCEH while weather data (Had-UK grid) can be queried from the CEDA archive. Unpublished trait data can be requested from the authors. Source data are provided with this paper.

Research involving human participants, their data, or biological material

Policy information about studies with human participants or human data. See also policy information about sex, gender (identity/presentation), and sexual orientation and race, ethnicity and racism.

Reporting on sex and gender	*Use the terms sex (biological attribute) and gender (shaped by social and cultural circumstances) carefully in order to avoid confusing both terms. Indicate if findings apply to only one sex or gender; describe whether sex and gender were considered in study design; whether sex and/or gender was determined based on self-reporting or assigned and methods used.*<br>*Provide in the source data disaggregated sex and gender data, where this information has been collected, and if consent has been obtained for sharing of individual-level data; provide overall numbers in this Reporting Summary. Please state if this information has not been collected.*<br>*Report sex- and gender-based analyses where performed, justify reasons for lack of sex- and gender-based analysis.*
Reporting on race, ethnicity, or other socially relevant groupings	*Please specify the socially constructed or socially relevant categorization variable(s) used in your manuscript and explain why they were used. Please note that such variables should not be used as proxies for other socially constructed/relevant variables (for example, race or ethnicity should not be used as a proxy for socioeconomic status).*<br>*Provide clear definitions of the relevant terms used, how they were provided (by the participants/respondents, the researchers, or third parties), and the method(s) used to classify people into the different categories (e.g. self-report, census or administrative data, social media data, etc.)*<br>*Please provide details about how you controlled for confounding variables in your analyses.*
Population characteristics	*Describe the covariate-relevant population characteristics of the human research participants (e.g. age, genotypic information, past and current diagnosis and treatment categories). If you filled out the behavioural & social sciences study design questions and have nothing to add here, write "See above."*
Recruitment	*Describe how participants were recruited. Outline any potential self-selection bias or other biases that may be present and how these are likely to impact results.*
Ethics oversight	*Identify the organization(s) that approved the study protocol.*

Note that full information on the approval of the study protocol must also be provided in the manuscript.

Field-specific reporting

Please select the one below that is the best fit for your research. If you are not sure, read the appropriate sections before making your selection.

☐ Life sciences ☐ Behavioural & social sciences ☒ Ecological, evolutionary & environmental sciences

For a reference copy of the document with all sections, see nature.com/documents/nr-reporting-summary-flat.pdf

Ecological, evolutionary & environmental sciences study design

All studies must disclose on these points even when the disclosure is negative.

Study description	Building on insect occurrence data (1252 species), we train trait-based models to predict species-level probability of occurrence across GB. With those models we identify trends in insect occupancy, the main environmental drivers behind those trends, as well as the key response traits mediating these drivers' effects. These model explanations, along with identified trends in trait assemblage, present an interesting picture of the biodiversity shifts seen in the country over the last 3 decades.
Research sample	The data used here is extensive and fall in three groups:<br>- occupancy data from 9 insect groups, either from structured recording schemes (butterflies, moths), or unstructured 'opportunistic' single species reporting (odonates, orthopterans, carabids, bees, wasps, ladybirds, hoverflies)<br>- response trait data for 8 of the 9 groups (all but wasps)<br>- environmental data (drivers) for weather (BIOCLIM19 from precipitation and temperature in the Had-UK grid 1km), for land-cover (proportion, diversity, contiguity from the UKCEH land-cover maps 25m), and topological static layer (terrain, distance to sea, river network)
Sampling strategy	Sampling occurred across the country over three decades. It is organised by national schemes with the additional support of citizen scientist. The resulting contingency tables (presence-absence aggregated at 1km2) on which the model are trained present sample sizes ranging from 1668 (for the moths, times 435 species) to 34146 (for the odonates, times 41 species). Those are large enough sample sizes that we can afford 50/50 train-test split for robust assessment of model performance. Risk of sampling bias are addressed within the study.
Data collection	Data was collected through structured recording schemes, as well as in unstructured opportunistic records by citizen scientists.
Timing and spatial scale	Insect data was collected from 1990 to 2019. Predictions are provided for years 1990 to 2020. The spatial extent is Great Britain.

Data exclusions	We excluded species that featured less than 30 observations across the 30 years of recording. We also excluded species for which response traits were not informed, except for wasps that we addressed wholly without trait data.
Reproducibility	The code is provided for reproducibility. However, a lot of data are not ours to offer and must be queried directly to the data managers of the respective recording schemes and trait data bases. The full data needed for training, explaining and predicting is given for one insect group, the moths, as this data is ours to share.
Randomization	Model training is done via ensembling in which the train-test split are randomised across 20 replicates to estimates (epistemic, aka data) uncertainty in predictions. Uncertainty estimation allowed to conservatively highlights trends, drivers and trait mediation throughout the study.
Blinding	Half the samples were retained for predictive performance assessment.

Did the study involve field work? ☐ Yes ☒ No

Reporting for specific materials, systems and methods

We require information from authors about some types of materials, experimental systems and methods used in many studies. Here, indicate whether each material, system or method listed is relevant to your study. If you are not sure if a list item applies to your research, read the appropriate section before selecting a response.

Materials & experimental systems

n/a	Involved in the study
☒	☐ Antibodies
☒	☐ Eukaryotic cell lines
☒	☐ Palaeontology and archaeology
☒	☐ Animals and other organisms
☒	☐ Clinical data
☒	☐ Dual use research of concern
☒	☐ Plants

Methods

n/a	Involved in the study
☒	☐ ChIP-seq
☒	☐ Flow cytometry
☒	☐ MRI-based neuroimaging

Plants

Seed stocks	*Report on the source of all seed stocks or other plant material used. If applicable, state the seed stock centre and catalogue number. If plant specimens were collected from the field, describe the collection location, date and sampling procedures.*
Novel plant genotypes	*Describe the methods by which all novel plant genotypes were produced. This includes those generated by transgenic approaches, gene editing, chemical/radiation-based mutagenesis and hybridization. For transgenic lines, describe the transformation method, the number of independent lines analyzed and the generation upon which experiments were performed. For gene-edited lines, describe the editor used, the endogenous sequence targeted for editing, the targeting guide RNA sequence (if applicable) and how the editor was applied.*
Authentication	*Describe any authentication procedures for each seed stock used or novel genotype generated. Describe any experiments used to assess the effect of a mutation and, where applicable, how potential secondary effects (e.g. second site T-DNA insertions, mosiacism, off-target gene editing) were examined.*
